# Effects of dopamine D1 modulation of the anterior cingulate cortex in a fear conditioning procedure

**DOI:** 10.1016/j.pnpbp.2015.08.015

**Published:** 2016-02-04

**Authors:** M.A. Pezze, H.J. Marshall, A. Domonkos, H.J. Cassaday

**Affiliations:** School of Psychology, University of Nottingham, United Kingdom

**Keywords:** Dopamine D1, Anterior cingulate, Trace conditioning, Contextual conditioning, Rat

## Abstract

The anterior cingulate cortex (AC) component of the medial prefrontal cortex (mPFC) has been implicated in attention and working memory as measured by trace conditioning. Since dopamine (DA) is a key modulator of mPFC function, the present study evaluated the role of DA receptor agents in rat AC, using trace fear conditioning. A conditioned stimulus (CS, noise) was followed by an unconditioned stimulus (US, shock) with or without a 10 s trace interval interposed between these events in a between-subjects design. Conditioned suppression of drinking was assessed in response to presentation of the CS or an experimental background stimulus (flashing lights, previously presented for the duration of the conditioning session). The selective D1 agonist SKF81297 (0.05 μg/side) or D1 antagonist SCH23390 (0.5 μg/side) was administered by intra-cerebral microinfusion directly into AC. It was predicted that either of these manipulations should be sufficient to impair trace (but not delay) conditioning. Counter to expectation, there was no effect of DA D1 modulation on trace conditioning as measured by suppression to the noise CS. However, rats infused with SKF81297 acquired stronger conditioned suppression to the experimental background stimulus than those infused with SCH23390 or saline. Thus, the DA D1 agonist SKF81297 increased conditioned suppression to the contextual background light stimulus but was otherwise without effect on fear conditioning.

## Highlights

• Previous studies have shown the role of anterior cingulate in trace conditioning.
• DA D1 receptor agents were micro-infused into anterior cingulate and tested in a CER procedure.
• Neither SKF81297 nor SCH23390 impaired trace conditioning.
• Contextual fear conditioning was increased by treatment with SCH23390.

## 1 Introduction

There is good evidence that the medial prefrontal cortex (mPFC) is essential for the smooth running of a number of cognitive functions including selective and divided attention, short term working memory and behavioral flexibility (Arnsten, 2000, Granon and Poucet, 2000, Kolb, 1984). Neuromodulatory effects have also been identified: local depletion of dopamine (DA) in the mPFC can cause cognitive deficits similar to those produced by conventional lesions. For example, the catecholaminergic toxin 6-hydroxydopamine injected within mPFC impaired performance in spatial delayed alternation tasks in both rats (Simon, 1981) and primates (Brozoski et al., 1979). Moreover, this behavioral deficit was reversed by the administration of the DA precursor L-DOPA or the DA agonist apomorphine (Brozoski et al., 1979). Thus, these early results suggested that DA is a key modulator of mPFC function.

Subsequent research has upheld the hypothesis that mPFC DA modulates attention and memory functions (Abi-Dargham et al., 2002, Arnsten, 1998, Granon et al., 2000, Pezze et al., 2015, Sawaguchi and Goldman-Rakic, 1991, Williams and Goldman-Rakic, 1995, Zahrt et al., 1997). Additionally, there is mounting evidence that an optimal level of mPFC DA is key to the maintenance of these functions. For example, either too high or too low a level of D1 stimulation may impair working memory (Castner and Goldman-Rakic 2004, Zahrt et al., 1997) and attention (Granon et al., 2000, Pezze et al., 2014). In turn, the optimal level of DA depends on the demands of the task (Chudasama and Robbins, 2004, Granon et al., 2000).

In simple associative learning procedures, the demands of the task can be increased by the introduction of a time gap (trace interval) between signal (conditioned stimulus, CS) and outcome (unconditioned stimulus, US). With respect to psychological mechanisms, both attention and working memory have been proposed to be essential to allow bridging of the time gap between the CS and US in trace conditioning procedures. With respect to neural substrates, dorsolateral PFC and dorsal AC activation have been identified with the attentional and/or working memory processes which underpin formation of associations between stimuli which have been separated in time (Gilmartin et al., 2014). For example, electrophysiological evidence suggests that AC neurons are part of an early attention system important for the identification of behaviourally salient stimuli more generally as well as for the acquisition of trace conditioning (Weible et al., 2003). Consistent with a role in conditioning, long-term-potentiation (LTP) induction in the AC results in the upregulation of AMPA receptor glutamate subunits (Descalzi et al., 2012). Moreover, there is behavioral evidence to suggest that rodent AC is necessary for trace conditioning (Descalzi et al., 2012, Han et al., 2003).

Han and colleagues used an attentional procedure in which the introduction of a visual distractor was demonstrated to interfere with trace but not delay conditioning in mice. This finding is consistent with the importance of awareness and attention in trace but not delay conditioning (Clark et al., 2002, Kalmbach et al., 2009). In a follow-up experiment, *c-fos* expression showed that trace conditioning was correlated with increased neuronal activity in the AC (Han et al., 2003). Furthermore excitotoxic lesion of the (rostral) AC resulted in the disruption of trace but not delay conditioning. Thus, the authors concluded that trace but not delay conditioning requires attention and the AC (Han et al., 2003). Similarly, in the rabbit, lesions to the mPFC that included AC reduced trace eyeblink conditioning (though in this case the lesions were caudal and lesions to rostral PFC were without effect; Kronforst-Collins and Disterhoft, 1998). In sum, although interventions in mPFC more typically target the prelimbic sub-region, studies using a variety of methods to alter or measure neuronal activity suggest the importance of AC in trace conditioning (Kalmbach et al., 2009, Kronforst-Collins and Disterhoft, 1998, Hattori et al., 2014, Warble et al., 2000, Weible et al., 2003; see Cassaday et al., 2014, for review).

Whilst providing compelling evidence for the role of AC in trace conditioning, as well as mPFC DA D1 receptor transmission in attention and working memory, studies to date have not addressed the specific role of DA within the AC (Cassaday et al., 2014). We have recently reported evidence for DA D1 modulation of trace conditioning and this effect was mediated (at least in part) in mPFC, however whilst we compared the effects of DA D1 agonist in prelimbic and infralimbic sub-regions (Pezze et al., 2015), the role of the AC was not examined in this previous study (and the procedure was appetitive, see below).

The present study was conducted to test the hypothesis that D1 receptor transmission in the AC modulates associative learning over a trace interval. There were a number of differences from the fear conditioning procedure used in the study by Han et al. (2003) which used mice rather than rats, a lower intensity shock presented over a greater number of trials and measured freezing rather than lick suppression. However, the procedure adopted in the present study is highly suitable to examine trace conditioning in the rat, and with demonstrated sensitivity to the effects of indirect (non-selective) DA agonists (Horsley and Cassaday, 2007, Norman and Cassaday, 2003). This conditioned emotional response (CER) procedure measures the level of conditioned fear by quantifying the suppression of a motivated response, in this case licking, in rats conditioned with or without a 10 s trace interval.

Intra cerebral micro-infusions were used to deliver the D1 agonist SKF81297 or the D1 antagonist SCH23390 directly into the AC, to examine effects on trace versus delay conditioning. Additionally, the level of contextual conditioning was measured by determining the level of suppression to an experimental background stimulus which had been presented for the duration of the conditioning session.

## 2 Materials and methods

### 2.1 Subjects

Forty-eight experimentally naïve male Wistar rats were used (Charles Rivers, UK; weights on arrival in the range 121–240 g). They were housed four per cage, on a 12:12 h dark/light cycle, and on ad libitum food and water (up until 24 h before shaping, see below). After arrival, each rat was handled for approximately 10 min per day over the course of one week. Rats were surgically prepared (implanted with guide cannulae) over the course of the following week at mean weight 272 g (range 252-285 g). After surgery each rat had a minimum of seven days' recovery period. One rat died because of an anesthetic complication; then during the recovery period, two animals fell ill and had to be humanely killed (post mortems revealed that one rat had contracted meningitis, the other had blood on the brain and an enlarged heart). Thus, in total 45 rats went on to be tested in the CER procedure, for which they were water deprived 24 h prior. For the duration of the behavioral experiment, water access was provided every day for one hour in the home cages. All procedures were carried out in accordance with the principles of laboratory animal care, specifically the United Kingdom (UK) Animals Scientific Procedures Act 1986, Project License number: PPL 40/3716.

### 2.2 Stereotactic surgery

Animals were injected with antibiotics (Synulax, 0.01 ml/200 g) 24 h prior to surgery, and thereafter daily until the end of the experiment. Perioperative analgesia was also administered pre-operatively ((Rimadyl large animal solution) 1:9 dilution for injection at 0.2 ml/250 g, s.c.). The rats were then anesthetized through a mouthpiece using isoflurane, delivered in oxygen (introduction: 4–5%; maintenance: 1–3%) and were secured in a stereotaxic frame. Next, the scalp was cut open and the skull was exposed. Bregma and lambda were aligned horizontally and two holes were drilled (SR Foredom; D#66 high speed drill bit) above the left and right AC. The coordinates for the cannulae insertions were the following: + 1.9 mm anterior, ± 0.6 mm lateral from bregma, − 2.3 mm ventral from the skull surface (Paxinos and Watson, 1998). Guide cannulae (the ‘mouse’ model C235GS-5–1.2; Plastic Ones) consisting of a 5 mm plastic pedestal that held two 26 gauge metal tubes, 1.2 mm apart and projecting 4.5 mm from the pedestal, were inserted bilaterally into the AC through the holes drilled. Finally, the cannulae were fixed with dental acrylic and with four stainless steel screws. Just prior to suturing, lidocaine (2% *w*/*v*, South Devon Healthcare) was administered around the incision area. To prevent infection, double stylets (33 gauge; Plastic Ones) were placed into the cannulae and the guides were closed with a dust cap. All animals had a minimum of seven days recovery period before the beginning of the experiment.

### 2.3 Drug administration

Rats were semi-randomly allocated to experimental conditions and injected 10 min prior to conditioning with either sterile 0.9% saline (n = 16), SKF81297 (n = 15) or SCH23390 (n = 14). Drugs were administered bilaterally through injectors (Plastic Ones) that were inserted to the guides. The injector tips extended 0.5 mm below the guides into the AC and they were connected through polyethylene tubing to two 5 μl syringes fixed on a microinfusion pump (SP200IZ Syringe). All drugs were administered in a volume of 0.5 μl/side over one min. The injectors remained in place for another one min to allow absorption and diffusion. To confirm that the liquid was successfully infused to the brain, air bubble movements in the tubing were observed. The procedure was identical for SKF81297 (Sigma, Poole, UK), injected at a concentration of 0.05 μg/side and for SCH23390 (Sigma, Poole, UK), injected at a concentration of 0.5 μg/side. Ten min after the completion of the microinfusion procedures rats were transferred into the behavioral boxes where they underwent conditioning. Drug administration was restricted to the conditioning day of the procedure.

### 2.4 Histology

Following the completion of the experiment, animals were anesthetized with a lethal dose of pentobarbital sodium (1–1.5 ml Euthatal; sodium pentobarbitone, 200 mg/ml; Merial Animal Health Ltd.) and perfused transcardially with 100 ml of 0.9% saline followed by 100 ml of 4% formaldehyde solution in saline. Subsequently the brains were removed whole from the skull and post-fixed in jars containing 10 ml of 4% formaldehyde. After a minimum period of four weeks, brains were cut into 80 μm coronal sections on vibratome. Brain sections encompassing AC were placed on slides and stained with cresyl violet. The locations of the cannulae placements were assessed using a light microscope and mapped to the atlas of Paxinos and Watson (1998).

### 2.5 Apparatus

Four identical fully automated conditioning boxes were housed within sound-attenuating cases containing ventilation fans (Cambridge Cognition, Cambridge, UK). The conditioning boxes were steel (25 cm × 25 cm × 22 cm high) with a Plexiglas door (27 cm × 21 cm high), inset at the front. A waterspout was mounted on one wall, 5 cm above the floor and connected to a lickometer supplied by a pump. Licks were registered by a break in the photo beam within the spout, which also triggered water delivery of 0.05 ml per lick. The waterspout was illuminated when water was available. The CS was a 5 s mixed frequency (80 dB) noise, delivered by a loudspeaker set in the roof of each box, presented at a 0 s or 10 s trace interval. Three wall-mounted stimulus lights and the house light were set to flash on (0.5 s) and off (0.5 s) for the duration of the conditioning session, to provide a flashing mixed wavelength (white) light experimental background stimulus. Footshock of 1 s duration and 1 mA intensity provided the US. This was delivered via the grid floor (steel bars 1 cm apart) by a constant current shock generator (pulsed voltage: output square wave 10 ms on, 80 ms off, 370 V peak under no load conditions; MISAC Systems, Newbury, UK). Stimulus control and data collection was by an Acorn Archimedes RISC computer programmed in Basic with additional interfacing using an Arachnid extension (Cambridge Cognition).

### 2.6 Procedure

**Fig. 1:**
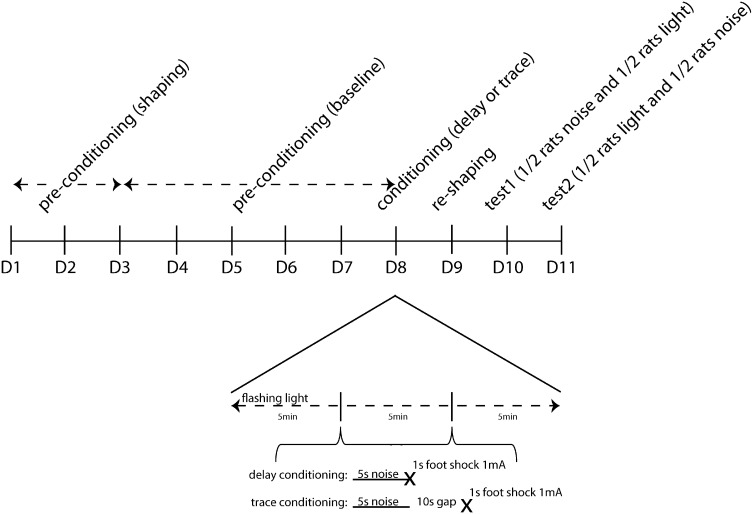
Timeline of the behavioral procedure from the first pre-conditioning day (D1) through to the second test day (D11) with a more detailed representation of the timing of events on the conditioning day (D8) shown in expanded format. On the conditioning day (D8) a 5 s noise CS was followed by a 1 s 1 mA footshock, immediately in the delay condition and after an interpolated 10 s time gap in the trace condition.

Water deprivation was introduced 1 day prior to shaping. The rats then had 1 h ad libitum access to water in their home cage after each of the procedural stages described below. This home cage access was in addition to any water drunk in the conditioning boxes (available from the apparatus waterspout on all days of the procedure apart from conditioning). Animals were trained, conditioned and tested in counterbalanced groups of four after 20–23 h of water deprivation, on consecutive days using the CER procedure illustrated in Fig. 1.

#### 2.6.1 Pre-conditioning to establish baseline lick responses

In order to initiate licking behavior, rats were first placed in the conditioning boxes in pairs (with their cage mates) and were shaped over 1 or more 15 min session until all drank from the waterspout. On a second shaping day, they were placed in the conditioning boxes individually to ensure that all were drinking freely. No data were recorded. Thereafter, animals were individually assigned to a conditioning box for the duration of the experiment (counterbalanced by experimental group). There then followed 5 days of pre-training, in which rats drank in their conditioning boxes for 15 min each day (timed from first lick). The licking spout was illuminated throughout, but no other stimuli were presented. The dependent variables were latency to first lick and the number of licks made within the first 1 min box exposure (analyzed for day 5 only).

#### 2.6.2 Conditioning with footshock

No water was available within the boxes and the waterspouts were not illuminated. The US footshock was delivered following termination of the CS in each of 2 conditioning trials per conditioning session (of which there were 2). The first pairing of CS and UCS was presented after 5 min had elapsed, and the second pairing was 5 min after the first, followed by a further 5 min left in the apparatus. In the trace conditioned group, there was a 10 s inter-stimulus-interval (10 s ISI) between the noise (CS) and footshock (US). In the delay conditioned control group, there was no interval between the CS and US (0 s ISI). The flashing lights experimental background was presented for the duration of the conditioning session within which the two conditioning trials took place. In the absence of licking, there were no behavioral measures to record.

#### 2.6.3 Reshaping after footshock

On the day following conditioning, animals were reshaped, following the same procedure as was used on the fifth day of pre-conditioning. This both re-established licking after conditioning and provided a measure of contextual conditioning, reflected in the extent to which licking was suppressed in the conditioning boxes. The dependent variables were latency to first lick and the number of licks made within the first 1 min box exposure.

#### 2.6.4 CER tests

Conditioned suppression to the experimental stimuli was tested in a counterbalanced order. On the first test day, 24 h after reshaping and 48 h after conditioning, the animals were placed in the conditioning boxes and presented with the noise CS or flashing light background stimulus. Water was available throughout the test and the waterspout was illuminated. Once the animals had made 50 licks, the CS was presented for 15 min. On the second test day, 72 h after conditioning, the alternate flashing lights background or noise CS was presented after 50 licks and continued for 15 min. The latency to make 50 licks in the absence of the CS or background stimulus (the A period, timed from the first lick made in each box) provided a measure of any individual variation in baseline licking. This was compared with the time taken to complete 50 licks following stimulus onset (B period) in a suppression ratio (A/(A + B)) to assess the level of conditioning, adjusted for any individual variation in drink rate.

### 2.7 Design and analysis

There were 6 experimental groups run in a 2 × 3 independent factorial design: trace condition at levels 0 s or 10 s and infusion at levels (saline, SKF81297 or SCH23390). The dependent variables were preconditioning drink latencies and the number of licks made during the first 1 min of the 5th session (to check for differences by experimental condition-to-be), reshaping drink latencies and the number of licks made during the first 1 min of the session (to assess contextual conditioning to the box cues), suppression ratios and the number of licks made during the first 1 min of stimulus presentation (to assess the levels of conditioning to the CS and experimental background stimulus). Significant main effects and interactions were further explored by Fisher's LSD test. Non-significant effects on baseline drinking are not reported.

## 3 Results

**Fig. 2:**
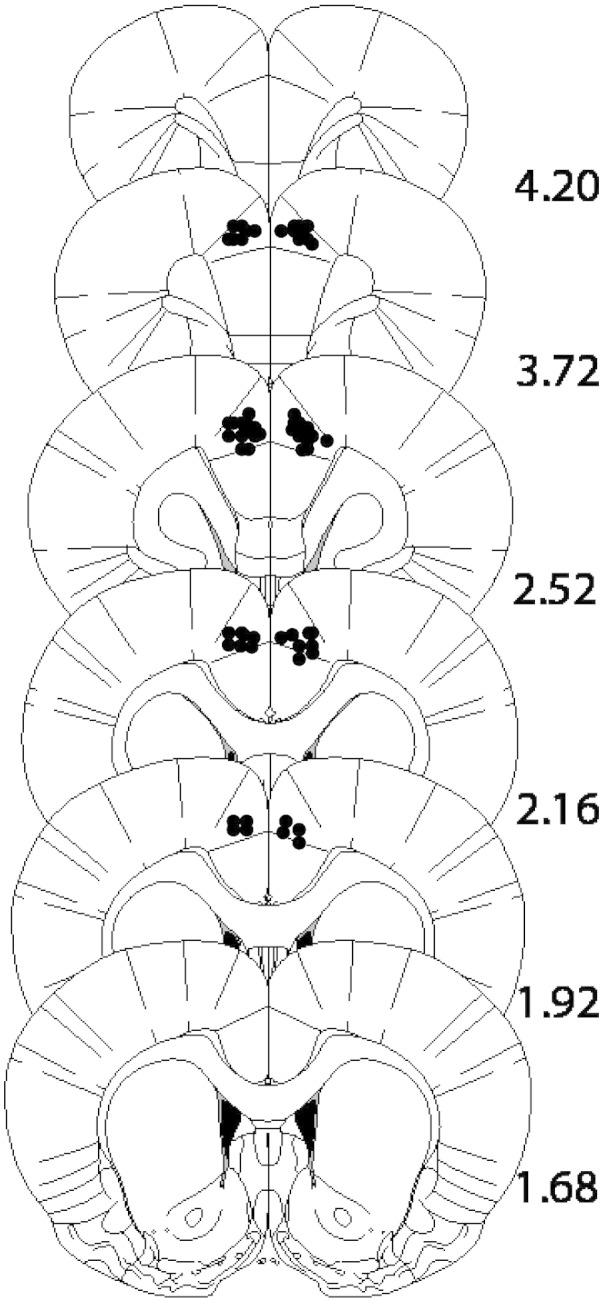
Approximate locations of infusion cannula tips, in the anterior cingulate of the mPFC. The placements for 3 animals could not be fully verified due to a technical problem with the histology procedures but there was no evidence to suggest that the injections were misplaced. Placements of the 41 brains which could be verified histologically are shown on coronal plates adapted from Paxinos and Watson (1998), with numbers indicating distance from bregma in millimeters.

The placements for 3 animals could not be fully verified due to a technical problem with the histology procedures but there was no evidence to suggest that the injections were misplaced. Fig. 2 shows the approximate locations of infusion cannula tips, in the AC of the mPFC. The final group sizes were as follows: 0 s-saline (n = 8), 0 s-SKF (n = 7) and 0 s-SCH (n = 7); 10 s-saline (n = 8), 10 s-SKF (n = 8) and 10 s-SCH (n = 7).

### 3.1 Pre-conditioning — baseline licking

On the final day of preconditioning, there was no effect of trace, *F*(1,39) = 1.589, *p* = 0.215, or infusion condition-to-be, *F*(2,39) = 0.708, *p* = 0.499, on the latency to start drinking. Neither was there any effect on the 1 min licks by trace, *F*(1,39) = 2.063, *p* = 0.159, or infusion condition-to-be, *F*(2,39) = 0.101, *p* = 0.904. Therefore, the rats' semi-random allocations resulted in experimental groups which were well matched for baseline drinking.

### 3.2 Reshaping — contextual conditioning

**Fig. 3:**
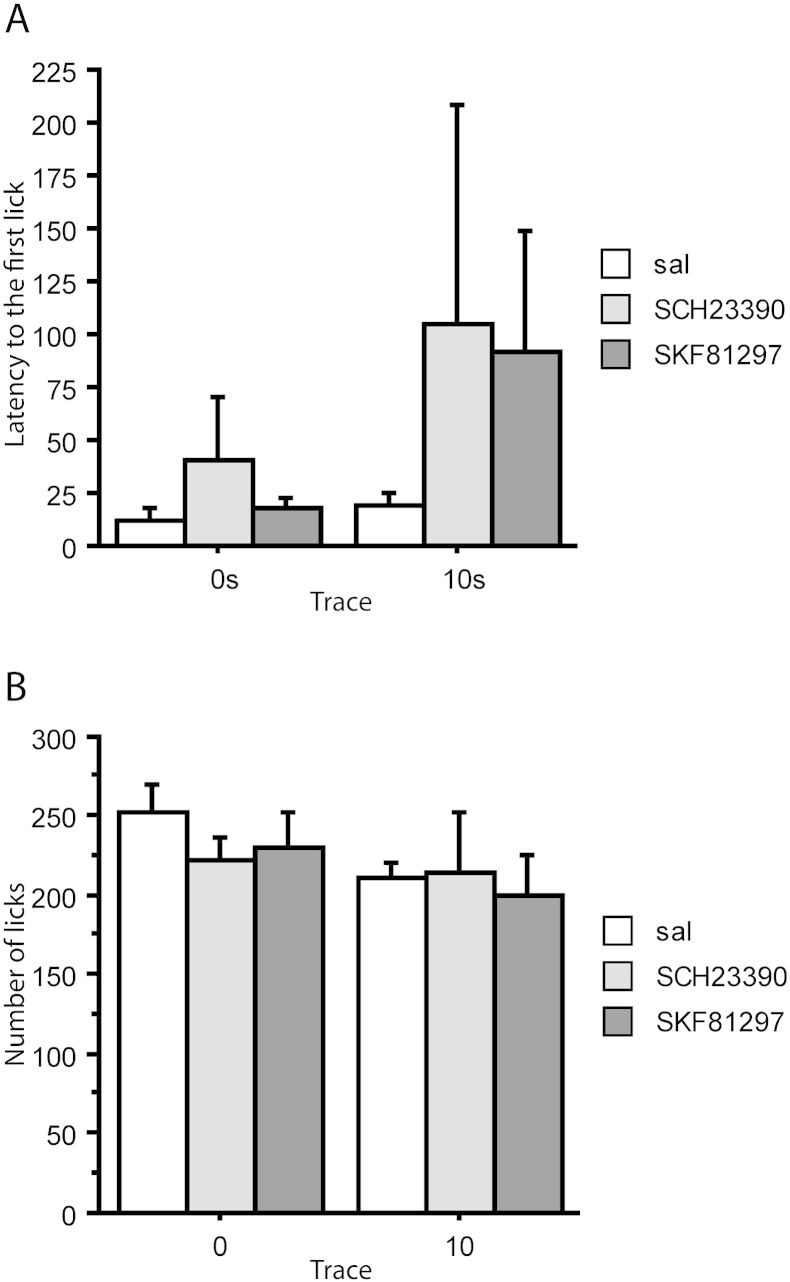
Box suppression at reshaping in 0 s and 10 s conditioned groups after conditioning under saline (sal), SKF81297 or SCH23390 infusions into anterior cingulate. The group sizes were 0 s-saline (n = 8), 0 s-SKF (n = 7) and 0 s-SCH (n = 7); 10 s-saline (n = 8), 10 s-SKF (n = 8) and 10 s-SCH (n = 7). (A) Mean latencies to drink. (B) Mean number of licks in the first 1 min. Error bars represent the standard error of the mean.

On the reshaping day after conditioning, there was no effect of trace condition, *F*(1,39) = 1.536, *p* = 0.223, and no effect of infusion, *F*(2,39) = 0.762, *p* = 0.473, on the latency to start drinking (Fig. 3A). Similarly, there was no difference on the 1 min licks by either trace, *F*(1,39) = 2.096, *p* = 0.156, or infusion condition, *F*(2,39) = 0.324, *p* = 0.725 (Fig. 3B). Thus there was no indication that the level of contextual conditioning – as measured by box suppression – was influenced by either trace condition (for the target CS) or by infusion with SKF81297 or SCH23390. Moreover, there was no interaction between trace and infusion condition, maximum *F*(2,39) = 0.300, *p* = 0.743.

### 3.3 CER tests

There was no overall effect of test order on suppression to the noise or light as measured by the suppression ratios or min 1 licks, maximum *F*(1,33) = 2.824, *p* = 0.102. No other effects involving test order were significant, maximum *F*(1,33) = 3.404, *p* = 0.074, for the interaction between trace condition and test order on the noise suppression ratio measure.

#### 3.3.1 CS (noise)

**Fig. 4:**
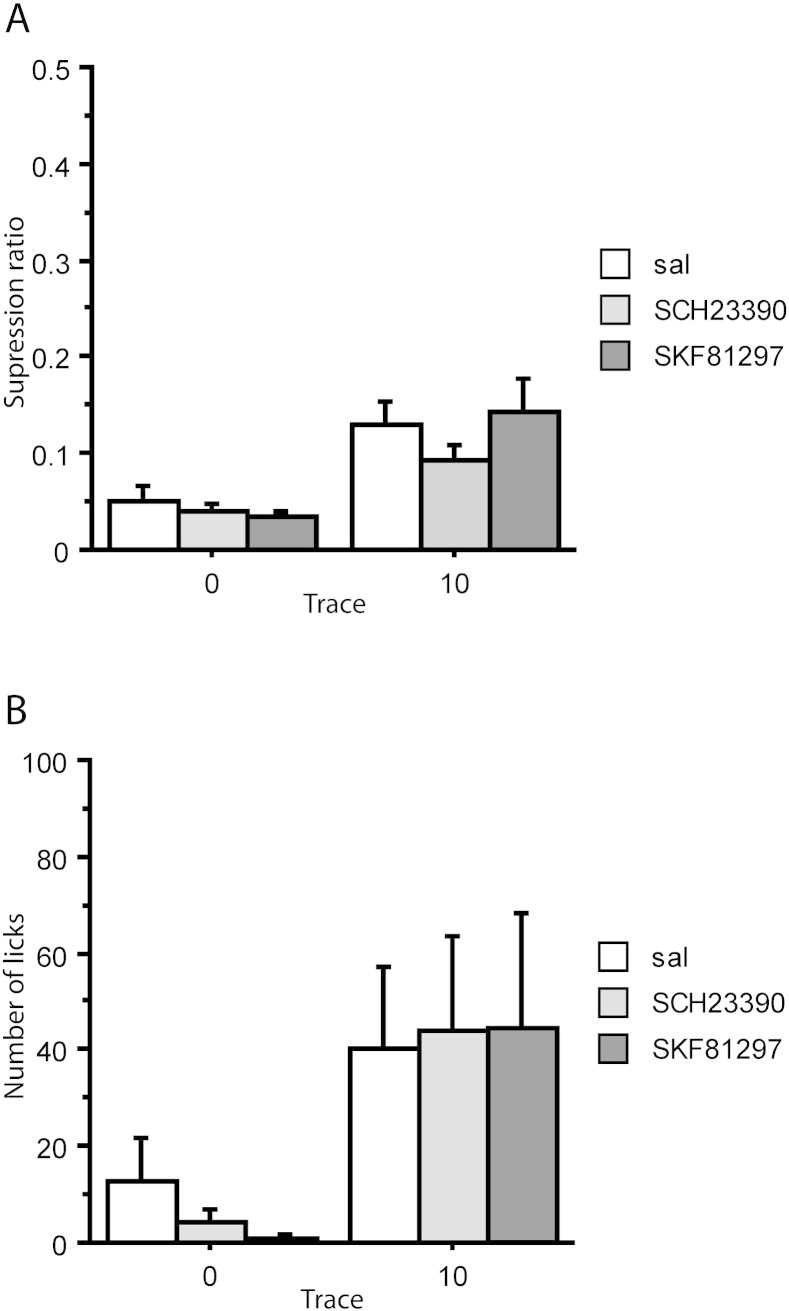
Conditioned suppression to the noise CS in 0 s and 10 s conditioned groups after conditioning under saline (sal), SKF81297 or SCH23390 infusions into anterior cingulate. The group sizes were 0 s-saline (n = 8), 0 s-SKF (n = 7) and 0 s-SCH (n = 7); 10 s-saline (n = 8), 10 s-SKF (n = 8) and 10 s-SCH (n = 7). (A) Mean suppression ratios. (B) Mean number of licks in the first 1 min. Error bars represent the standard error of the mean.

ANOVA of both the suppression ratio and 1 min licks measures showed the expected main effect of trace, *F*(1,39) = 21.028, *p* < 0.001, and *F*(1,39) = 8.989, *p* = 0.005, respectively. However, there was no effect of infusion, maximum *F*(2,39) = 0.804, *p* = 0.455; neither was there any interaction between infusion and trace, maximum *F*(2,39) = 0.767, *p* = 0.471 (Fig. 4). Overall, rats conditioned at 0 s were strongly suppressed to the noise CS whereas the introduction of the 10 s trace interval resulted in attenuated conditioning. Counter to prediction, the level of trace conditioning was not influenced by infusion with either SKF81297 or SCH23390.

#### 3.3.2 Experimental background (flashing lights)

**Fig. 5:**
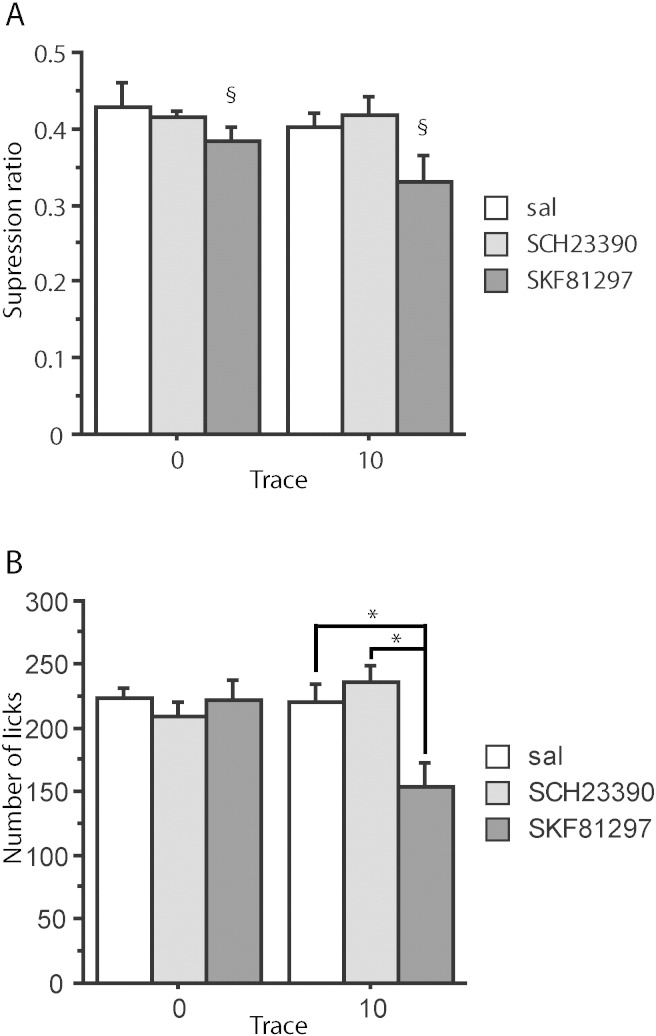
Conditioned suppression to the flashing lights background stimulus in 0 s and 10 s conditioned groups after conditioning under saline (sal), SKF81297 or SCH23390 (SCH) infusions into anterior cingulate. The group sizes were 0 s-saline (n = 8), 0 s-SKF (n = 7) and 0 s-SCH (n = 7); 10 s-saline (n = 8), 10 s-SKF (n = 8) and 10 s-SCH (n = 7). (A) Mean suppression ratios. §Denotes a significant effect of infusion, as confirmed by Fischer's LSD test, *p* < 0.05 (Fig. 5A). (B) Mean number of licks in the first 1 min. Error bars represent the standard error of the mean. *Comparison lines are used to show difference in the effect of infusion by trace condition, as confirmed by Fischer's LSD test, *p* < 0.05 (Fig. 5B).

ANOVA of the suppression ratios showed a main effect of infusion, *F*(2,39) = 3.583, *p* = 0.037, but no effect of trace condition, *F*(1,39) = 1.461, *p* = 0.234, nor any interaction between trace condition and infusion, *F*(2,39) = 0.572, *p* = 0.569 (Fig. 5A). The main effect of infusion arose because conditioning to the contextual light stimulus was overall increased under SKF81297, relative to both the saline, *p* = 0.020, and the SCH23390-treated group, *p* = 0.023, whereas there was no difference between the saline and SCH23390-treated group, *p* = 0.984.

Similarly, the 1 min licks analysis showed no effect of trace condition, *F*(1,39) = 1.500, *p* = 0.228, and in this case both a main effect of infusion, *F*(2,39) = 4.057, *p* = 0.025, as well as an interaction between trace condition and infusion, *F*(2,39) = 5.897, *p* = 0.006. Fig. 5B shows that the main effect of drug arose because, consistent with the findings on the suppression ratio measure, rats conditioned under SKF81297 drank less than both saline- SCH23390-treated rats, both *p* = 0.012. It can also be seen from Fig. 5B that this effect was carried by the 10 s trace conditioned group (hence the significant interaction on the licks measure). Specifically, rats trace conditioned under SKF81297 drank less than rats trace conditioned under saline, *p* = 0.001, or SCH23390, *p* < 0.001, whereas there was no difference between the saline and the SCH23390-treated trace conditioned groups, *p* = 0.399. There were no significant differences by drug treatment amongst the delay (0 s) conditioned groups, minimum *p* = 0.469.

## 4 Discussion

The present experiment evaluated the effect of DA D1 receptor modulation on cognitive function in the AC as assessed by trace conditioning measured in a CER (fear conditioning) procedure in which the level of contextual conditioning is also routinely assessed. As expected, rats conditioned over a 10 s trace interval showed less conditioned fear than the 0 s delay conditioned controls. Counter to expectation, there was no evidence that intra-AC infusions of either the D1 agonist SKF81297 or the D1 antagonist SCH23390 had any effect on trace conditioning. The level of delay conditioning in the 0 s control groups was similarly unaffected by these treatments. However, as discussed below, DA D1 modulation in AC was not without effect in that the level of contextual conditioning to an experimental background stimulus was increased under SKF81297. There was no such effect on suppression to the contextual cues provided by the conditioning boxes.

### 4.1 The relationship between D1 activity and cognitive function

Cognitive-behavioral performance is known to depend on an optimal level of mPFC dopaminergic activity. In other words, both too little and too much DA stimulation can impair mPFC-mediated functions following an inverted U shaped function (Abi-Dargham et al., 2002, Arnsten, 1998, Castner and Goldman-Rakic 2004, Chudasama and Robbins, 2004, Granon et al., 2000, Pezze et al., 2014, Robbins, 2000, Williams and Goldman-Rakic, 1995, Zahrt et al., 1997). Indeed this non-linearity was the justification for comparing the effects of both a D1 agonist and antagonist in the present study, rather than comparing the effects of different doses of agonist for example.

Previous studies have reported doses of SKF81297 and SCH23390 which are effective for DA modulation (Chudasama and Robbins, 2004, Granon et al., 2000, McGregor and Roberts, 1995, Ramos et al., 2005, Runyan and Dash, 2004, Seamans et al., 1998, Zahrt et al., 1997). For example, Zahrt et al. (1997) reported impaired spatial working memory after bilateral mPFC infusion of SKF81297 at 0.1 μg but not 0.01 μg. This effect was reversible by treatment with SCH23390 and was attributed to supranormal D1 receptor stimulation (Zahrt et al., 1997). Chudasama and Robbins (2004) examined the effects of mPFC SKF81297 at 0.01, 0.06 and 0.3 μg, in this case using the serial reaction time task. In this study both of the higher doses improved attentional performance, only the lower 0.01 μg dose was ineffective (Chudasama and Robbins, 2004). The present experiment infused SKF81297 at 0.05 μg/side, this dose being selected as in the mid-range of doses tested by Zahrt et al. (1997) and very close to one of the effective doses used by Chudasama and Robbins (2004). Because of the relatively small size of the AC, the concentration of SCH23390 was lower than that used in micro-infusion studies of other brain regions (Pezze and Bast, 2012, Heath et al., 2015) but suitable for use in mPFC (Seamans et al., 1998, McGregor and Roberts, 1995, Runyan and Dash, 2004). It remains possible, however, that effects on trace conditioning might have been demonstrated using different concentrations of D1 agonist or antagonist, if either of the doses selected was too low or too high.

### 4.2 Cannulae placements

The rationale for the present study was based on a previous rodent lesion study which demonstrated that the acquisition of trace conditioning depends on the AC (Han et al., 2003). The results obtained in rabbits using eyeblink conditioning procedures suggest a slightly different locus of effects. Specifically, Weible and co-workers investigated the role of different subdivisions of the mPFC in the rabbit and showed that whilst lesions of the rostro-medial PFC including the rostral AC were without effect on the acquisition of trace conditioning, lesions to the caudal part of mPFC including the caudal AC completely abolished the acquisition of trace conditioning (Weible et al., 2000). This group further reported that recordings of single neuron activity in the caudal AC during the training session of a trace conditioning procedure suggest that the causal AC is a part of an attentional mechanism for detecting coincidence between temporally-related stimuli (Weible et al., 2003). Taken together, the studies of Warble et al., 2000, Weible et al., 2003, suggest that the caudal part of the AC is critical for the acquisition of trace conditioning while there is relatively less evidence for role of its rostral part (but see Han et al., 2003). In our study, although the surgery coordinates targeted the middle part of the AC, histological examination revealed that our cannulae placements were in fact located relatively rostrally within the AC. Thus this difference in placement may account for the lack of effect on trace conditioning after infusions of either the D1 receptor agonist SKF81297 or the antagonist SCH23390. Alternatively, the difference in outcomes maybe attributable to the use of eyeblink procedures in the aforementioned rabbit studies: cerebellum is known to be a particularly important neural substrate for eyeblink conditioning, irrespective of whether the procedure in use is trace or delay conditioning; additionally, eyeblink procedures typically use very short (ms) trace intervals (Chen et al., 2014).

### 4.3 The role of other neurotransmitters in AC

DA D1 receptors are not the only available target to modulate AC function. Therefore another potential reason for the lack of effect on trace conditioning of either SKF81297 or SCH23390 in AC is that DA D1 was not the most suitable target (Arnsten, 1998, Robbins and Roberts, 2007). Dysfunction of DA neurons within the mPFC has been linked to a variety of cognitive dysfunctions, including attention deficit hyperactivity disorder (ADHD) (del Campo et al., 2011) and schizophrenia (Abi-Dargham et al., 2002), as well as age-related memory decline (Castner and Goldman-Rakic, 2004). However, the catecholamine family of neurotransmitters includes noradrenalin (NA) as well as DA and NA has been implicated in many of the same disorders as DA, including Parkinson's disease, ADHD and schizophrenia (Arnsten, 1998). Thus, the very selectivity of the interventions employed in the present study may account for their lack of effect on trace conditioning which has been reported to be impaired after excitotoxic lesions in AC (Han et al., 2003). Indeed, reversible inactivation produced using muscimol in AC might provide a better method to reproduce the effects of excitoxic lesions with the temporal resolution afforded by microinfusion as distinct from lesion methods.

### 4.4 Increased contextual conditioning under SKF81297

It might at first sight seem surprising that SKF81297 should increase conditioned suppression to one experimental stimulus (the flashing lights background) but not another (the noise CS at either 0 s or 10 s trace interval), particularly given the evidence that discrete cue fear conditioning relies on mPFC (Johansen and Fields, 2004, Knight et al., 2004, Morgan and LeDoux, 1995, Morrow et al., 1999). As was inevitably the case for the other contextual cues (provided by the conditioning boxes), the identities of the stimuli used for target and contextual background stimulus were not counterbalanced as trace conditioning to the noise was of primary interest (Han et al., 2003). In previous studies, Wistar rats showed no differences by the stimulus modality of the experimental background stimulus in this CER procedure (Cassaday et al., 2001, Norman and Cassaday, 2004). Moreover, as shown in Fig. 5, the observed increase in suppression under SKF81297 was more evident in the trace group which means that modality per se is an unlikely account of this effect.

There is independent evidence for dissociable effects on conditioning to contextual versus discrete cues, of the kind provided by the 15 min light background and the 5 s noise CS, respectively (Cassaday et al., 2001, Hirsh, 1974, Phillips and Le Doux, 1992, Selden et al., 1991, Winocur et al., 1987). Moreover, a disruption of neuronal activity in the mPFC has been shown to impair both trace and delay contextual fear conditioning (Gilmartin and Helmstetter, 2010), and selective blockade of the NR2B subunit of the NMDA receptor in the AC reduced LTP and impaired contextual fear conditioning (Zhao et al., 2005). Thus, the increased conditioning seen to the extended (15 min) background but not to the discrete (5 s) CS may reflect the same kind of dissociation now seen under SKF81297 in AC. Consistent with our data, these findings also suggest that the dorsal part of the mPFC contributes to the formation of contextual fear memory (Zhao et al., 2005).

Our results suggest that DA D1 receptor transmission within the AC may play a role in the formation of contextual fear conditioning as has previously been demonstrated in nucleus accumbens (Albrechet-Souza et al., 2013). However, in the latter study contextual fear conditioning was increased by treatment with SCH23390, rather than an agonist as in the present study (Albrechet-Souza et al., 2013). Interestingly a positive modulation of D1 receptor on the induction and maintenance of LTP is well characterised in the mPFC (Jay, 2003, El-Ghundi et al., 2007). Together these lines of evidence suggest that the formation of contextual fear memory may rely on the modulation of LTP by the D1 receptor. If the increased conditioning to the experimental background stimulus observed in the present study does indeed reflect increased contextual conditioning then suppression to the cues provided by the conditioning boxes (measured at reshaping) would also be expected to be increased. However, the box cues were effectively pre-exposed (over the course of pre-conditioning) and such pre-exposure would be predicted to reduce their associability through latent inhibition (Lubow and Moore, 1959). Moreover, the flashing lights background might be expected to overshadow the contextual cues provided by the conditioning boxes (Cassaday, 2010, Cassaday and Thur, 2015). Likewise, the fact that this effect was more pronounced in the trace conditioned group points to the importance of the attentional demands of the task, because for the trace conditioned group there is more potential for the light background stimulus to overshadow the noise CS (Marlin, 1981, Tanner et al., 1987).

### 4.5 AC role in emotional regulation

Although there is some evidence for the role of the AC sub-region of the mPFC in trace conditioning (Gilmartin et al., 2014, Han et al., 2003, Kronforst-Collins and Disterhoft, 1998, Warble et al., 2000) – compared with prelimbic and infralimbic sub-regions – there is relatively little evidence for the role of AC in cognitive processes. In contrast, there is a vast body of evidence for its role in emotional regulation (Li et al., 2014, VanElzakker et al., 2014). In particular the AC has a role in processing pain perception (Yan et al., 2012) and potential threat assessment (Fiddick, 2011), both of which could potentially confound the present study. However, there was no effect on fear conditioning to the noise CS in either the trace or delay condition and the increase in contextual conditioning (under SKF81297) was seen only to the experimental background stimulus (there was no such increase in suppression to the cues provided by the conditioning boxes).

### 4.6 Conclusions and implications

Contrary to expectation, there was no effect of DA D1 modulation on trace conditioning as measured by suppression to the noise CS, nor was there any effect on contextual conditioning to the box cues as measured at reshaping. However, there was an increase in conditioning to an experimental background stimulus under SKF81297 which is consistent with an effect on conditioning to context, albeit the modality, relative salience and/or the familiarity of contextual cues may be important determinants of whether such effects are seen. Increased contextual fear conditioning under SKF81297 is broadly consistent with the role of AC in emotional processing and in particular disorders of emotional processing such as PTSD (Li et al., 2014, VanElzakker et al., 2014) in which the role of contextual triggers is well documented (Ehlers and Clark, 2000). The results of the present study suggest that DA D1 receptor activation in AC may increase contextual conditioning, particularly in the absence of more reliable (discrete cue) predictors in that (on the first 1 min licking measure of conditioned suppression) this effect was more evident in the trace condition.

## Conflicts of interest

None.
